# The pathogenesis of foot-and-mouth disease virus: current understandings and knowledge gaps

**DOI:** 10.1186/s13567-025-01545-5

**Published:** 2025-06-16

**Authors:** Carolina Stenfeldt, Michael Eschbaumer, John Humphreys, Gisselle N. Medina, Jonathan Arzt

**Affiliations:** 1 Foreign Animal Disease Research Unit, U.S. Department of Agriculture-Agricultural Research Service, Plum Island Animal Disease Center, Greenport, NY USA; 2 College of Veterinary Medicine, Department of Diagnostic Medicine/Pathobiology, Kansas State University, Manhattan, KS USA; 3 Institute of Diagnostic Virology, Friedrich-Loeffler-Institut, Greifswald-Insel Riems, Germany; 4 Foreign Animal Disease Research Unit, U.S. Department of Agriculture-Agricultural Research Service, National Bio-and Agro Defense Facility, Manhattan, KS USA

**Keywords:** Foot-and-mouth disease, FMDV, FMD, pathogenesis, viral infection, virus, host response, transmission, FMDV carrier, neoteric infection

## Abstract

Foot-and-mouth disease (FMD) continues to be one of the most important diseases of livestock globally based upon both biological features and regulatory aspects. Few pathogens have had comparable impact on global livestock production and regulation of international trade in animal-derived products. The pathogenesis (interaction between pathogen and host) is central to the importance of the disease ranging from how the causal pathogen, FMD virus (FMDV), transmits between hosts and is maintained in populations. Key accomplishments over the last decade include description of the primary sites of infection in domestic species, delineating critical differences in temporo-anatomic progression in different host species and emphasizing that knowledge gained regarding FMDV pathogenesis in one host cannot necessarily be extrapolated and applied to a different host. Host responses to infection and viral genomics have been characterized with ever-increasing granularity. Yet, the numerous knowledge gaps that remain in understanding FMDV pathogenesis impede advancements in FMD control and eradication. For instance, it remains unclear if long-term asymptomatic FMDV carriers are biologically relevant (contagious) and the manner in which host genomics and transcriptomics affect pathogenesis during different phases of infection. The characterization of neoteric subclinical infection as a disease stage that is distinct from the persistent “FMDV carrier state” has emphasized the importance of sample collection from clinically unaffected animals for FMDV surveillance. Similarly, incorporating a phase of pre-clinical infectiousness in simulation modeling can dramatically improve prediction of FMD outbreaks in non-endemic regions. The outcome of FMDV infection with regards to viral persistence differs between host species as well as between individuals of the same species. Yet, we lack a satisfactory explanation of the host factors that drive the FMDV carrier state divergence. This review was based upon a gap-analysis workshop organized by the Global Foot-and-Mouth Disease Research Alliance (GFRA) in Buenos Aires, Argentina, in December of 2022. The purpose of this work is to summarize the current understanding of the distinct compartments of FMD pathogenesis with an emphasis on progress made within the last decade and present the critical knowledge gaps that continue to limit FMD control and eradication.

**Table 1:** **Summary of FMDV-host interactions and immune evasion strategies during early and late phases of infection.**

Type	Key findings	Mechanisms/Proteins involved	References
Innate Immune Response	• FMDV triggers TLR3, 7, 8, and other sensors like RIG-I and MDA5 • Production of IFN-I/III and pro-inflammatory cytokines (TNF-α, IL-1, IFN-γ) • pDCs are major IFN producers in cattle • NK cells critical but impaired in pigs	• IRF3/7, NF-κB, ISGs, NK cells production of IL-12, DC maturation • Endosome (TLRs) and cytosolic detection of FMDV (RIG-I and MDA5)	[119–126]
Viral Immune Evasion	• Capsid proteins: VP0 blocks IFN-β; VP2 induces autophagy; VP1 inhibits NF-κB, VP3 inhibits type I and II signaling • Non-structural proteins: Lpro cleaves eIF4G, TBK1, MAVS, NF-κB, G3BP1/2, ISG15 or Ub-conjugated substrates; 3 A inhibits IFN-β signaling; 3 C cleaves NEMO, STAT1, etc	• Host translation shut off (cap independent) • Modulation of cellular transcription (histone cleavage) • Modulation of IFN signaling pathways • Stress granule disruption (G3BP1/G3BP2) • Disruption of post-translational modifications (i.e., ubiquitination and Isgylation) • RLR/NLR (i.e., RIG-I, MAVS, MDA5) pathway inhibition	[128–154, 191]
Species-specific differences	• Cattle: robust systemic IFN and acute phase proteins • Pigs: variable IFN response; IL-10 mediated immunosuppression during acute infection	• Serum amyloid A, Haptoglobin, type I IFN • 3 A deletions affect virulence in cattle but not in pigs	[121, 127, 148–151, 155]
Adaptive Immunity	• Antibody response: rapid IgM to IgG/IgA response; may contribute to long-lasting humoral immunity in cattle • Mucosal Immunity: local IgA in FMD carrier state but fails to clear persistent virus (intracellular evasion)	• Serum transudation (early) versus local IgA (persistent phase) in oral and nasal secretions; exosomes may aid viral spread	[159–167]
Persistence	• Persistent infection may be linked to impaired cell mediated immunity • Exosomes may shield viruses from antibodies (similar to Hepatitis A virus)	• T cell-killing and death receptor pathways are impaired in FMDV carriers • Inhibition of T cell activation and promotion of Th2 polarization	[66, 77]

## Introduction

Foot-and-mouth disease (FMD), caused by FMD virus (FMDV; species: *Aphthovirus vesiculae*, genus: *Aphthovirus*, family: *Picornaviridae*) has been an impediment to livestock production for centuries. Many aspects of pathogenesis determine the importance of FMD, in particular the high contagiousness and broad range of wild and domestic hosts, including cattle, pigs, sheep, goats, and buffalo. The World Organization for Animal Health (WOAH) estimates that the disease currently circulates in over 70% of the global livestock population. FMD is endemic in vast areas of Africa and Asia, regionally endemic in South America whereas North America, Europe, and Australia have been kept mostly free of FMD through several decades by strict regulation on import of animals and animal products. FMD freedom in continental Europe was in early 2025 disrupted by incursions of two unrelated strains of FMDV serotype O affecting Germany as well as Hungary and Slovakia [1–3].

Foot-and-mouth disease in naïve animals is characterized by a period of fever coinciding with the occurrence of vesicular lesions (blisters) on the oral mucosa and areas of non-haired skin, including coronary bands, interdigital clefts, and teats [4, 5]. The severity of clinical FMD varies greatly depending both on intrinsic characteristics of the different virus strains, as well as on host genetics and immune status. Typical clinical syndromes range from debilitating lameness and anorexia, to barely noticeable oral blisters in otherwise unaffected animals. Fatal myocarditis is a relatively uncommon sequela that affects predominantly juvenile animals of various species [4]. FMDV can also cause two distinct forms of fully subclinical infections. Early-phase subclinical (neoteric) infections in all hosts may be associated with substantial virus shedding in oronasal secretions, whereas post-acute, long-term persistent infections which occur in ruminants, generally do not involve virus shedding (Reviewed in: [6]). Neoteric subclinical infections are most commonly seen in animals with acquired immunity, either from previous exposure or vaccination, whereas persistent FMDV infection occurs in ruminants subsequent to either clinical or subclinical infection, regardless of vaccination or immune status.

Due to the severe consequences of FMD outbreaks, access to international trade in animals and animal-derived products is generally dictated by country-level official FMD status. Thus, countries that are affected by FMD outbreaks suffer not only from direct impacts on animal health and production, but also from the financial implications of being excluded from export markets [7, 8]. Control of FMD by combination of vaccination and depopulation was successful in Europe, the Philippines and large parts of South America as well as many FMD-free countries that had an introduction of FMD. But control by vaccination is expensive and is only successful in combination with other control measures. For this reason, the Progressive Control Pathway for FMD (PCP-FMD) was developed by the Food and Agriculture Organization of the United Nations (FAO) and the European Commission for the Control of foot-and-mouth Disease (EuFMD) as a tool to assist low- and middle-income countries to gradually improve control of FMD. This program is intended to support the establishment of national FMD control programs that may eventually result in a WOAH -endorsed status as free of FMD.

Cattle are often considered the most relevant hosts, and are the best-studied amongst domestic livestock. However, there are important contributions of other livestock species in the epidemiology of this disease, and those contributions are determined by the variable pathogenesis across species. Recent research has demonstrated multiple differences in the pathogenesis and transmission of FMDV amongst common livestock species. Specifically, there are considerable differences in the anatomic sites of both initial and persistent infection, which affects susceptibility to infection via different exposure routes, as well as virus shedding and detection by different sampling techniques. Additionally, the combined characteristics of virus shedding and primary infection sites affect the probability of, and physical requirements for transmission within and between different host species. For example, the high permissibility of the bovine upper respiratory tract to infection means that cattle are susceptible to aerogenous spread of FMDV, whereas multiple experimental studies have shown a relative resistance of pigs to air-borne infection and a requirement for direct physical contact between animals for efficient transmission amongst pigs [9, 10].

The aim of this review is to provide an updated account of current knowledge of FMDV infection, host response, and transmission in commonly affected host species, and to summarize the relevant knowledge gaps that remain. The intention is to expand upon previous GFRA gap analysis reports, with a specific emphasis on relevant differences in pathogenesis and transmission dynamics amongst commonly affected livestock species. Overall, the global financial and socioeconomic impacts of FMD are considerable, both in countries striving to maintain a status as free of FMD, and in the large regions of the world in which FMD is endemic. A thorough understanding of the differences and similarities of FMD in different hosts and how these differences may affect detection and spread of infection is critical for optimal and prioritized disease control measures in areas affected by FMD.

## Molecular basis of FMDV pathogenesis obtained through in vitro investigation

**Figure 1:**
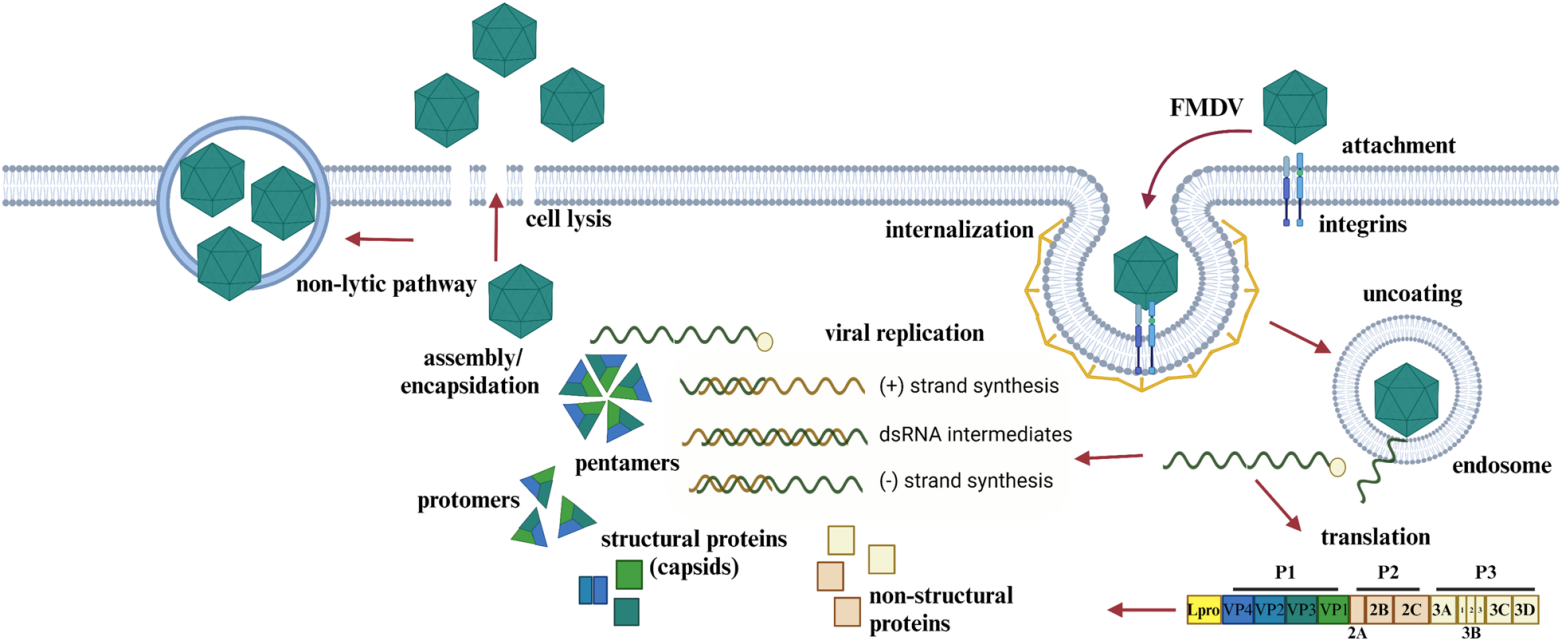
**Overview of FMDV life cycle**. The replication cycle of FMDV begins with the virus binding to cellular receptors (e.g., αVβ6, heparin sulfate), triggering internalization through clathrin-mediated endocytosis or alternative pathways. Acidification of the endosome facilitates uncoating, releasing viral RNA into the cytoplasm. The RNA is translated into a polyprotein, cleaved by viral proteases (Lpro, 3 Cpro), and processed into structural and non-structural proteins. Replication is suggested to occur in virus-induced membrane structures also known as replication organelles (ROs), where the RNA-dependent RNA polymerase (3D) synthesizes a complementary negative-strand (−) RNA that serves as a template for synthesis of new (+) RNA molecules. Capsid proteins assemble into protomers and pentamers and together with genomic viral RNA, assemble into infectious mature virions. Mature virions exit the host cell via cell lysis or via non-lytic mechanisms, such as extracellular vesicles (EVs). Image created in BioRender.

FMDV is a non-enveloped virus of approximately 30 nm in diameter, with a single-stranded genome of positive polarity (+) RNA of roughly 8400 nucleotides in length. FMDV initiates infection by binding to integrins on epithelial cells, specifically αVβ1, αVβ3, αVβ6, and αVβ8 (reviewed in [11]), which are cell surface receptors that facilitate cell adhesion and signaling. However, adapted strains of FMDV cultured under laboratory conditions have also been shown to interact with alternative receptors, such as heparan sulfate [12]. The cell binding typically occurs through a highly conserved and flexible RGD motif (Arg-Gly-Asp) located in the G-H loop of the VP1 protein (reviewed in [11]). The interaction with the receptors triggers the internalization of the virus via clathrin-coated pits or caveolae-mediated endocytosis [13, 14] (Figure 1), processes that can be enhanced by interactions between motor proteins (i.e., KIF5B) and the structural protein VP1 [15]. However, alternative methods of internalization have also been described, including micropinocytosis [16].

Once inside the cell, the acidification of endosomes triggers the uncoating or disassembly of the virus into pentameric subunits. This process releases the viral RNA and internal proteins, enabling the viral genome to exit into the cytoplasm through a pore in the endosomal membrane [17]. It is suggested that the hydrophobic properties of FMDV VP4, along with its myristoylation [18], play a crucial role in mediating membrane permeabilization and forming ion channels, thereby facilitating the exit of FMDV RNA from the endosome, although the precise mechanisms remain to be fully elucidated.

After the viral RNA enters the cytosol, it is translated into a large polyprotein, which is subsequently cleaved by viral proteases Lpro and 3 Cpro (Figure 1). The initial processing of the FMDV polyprotein yields four primary products: Lpro, P1 (capsid precursor P1-2 A), and two non-structural protein precursors, P2 (2BC) and P3 (3 AB1,2,3 CD). Although the specific role of replication organelles (ROs)—virus-induced membrane structures—has not been clearly defined for FMDV, replication by other picornaviruses occurs within these organelles [19]. In these ROs, the RNA-dependent polymerase 3D synthesizes multiple new viral positive-strand RNAs. This process involves the synthesis of a negative-strand copy of the incoming viral genome, generating a double-stranded RNA replication intermediates. The negative strand then serves as a template for production of new positive strands (Figure 1). Newly synthesized viral RNAs can either act as templates for further translation and replication or be encapsidated into new virions. The translation of the polyprotein can be aided by distinct chaperon proteins to enhance processing of P1 as described by Newman et al. [20]. While the encapsidation signals within the FMDV genome are not yet fully understood, recent research indicates that critical signals located in predicted secondary structures of the genome play a vital role in the interaction between FMDV RNA and capsid precursors [21]. This interaction is essential for the proper folding and packaging of the genome into the assembling capsid. FMDV particles are formed by the assembly of structural capsid proteins VP0, VP1, and VP3 into protomers and pentamers. Ultimately, the RNA-induced processing of VP0 into VP2 and VP4 results in the formation of mature virions [22, 23]. It is also important to note that during replication, certain viral non-structural proteins and precursors interact with RNA elements located in the 5′ and 3′ untranslated regions (UTRs) that flank the open reading frame of the FMDV genome. These interactions play a critical role in regulating viral replication and translation (reviewed in [24, 25]). Lastly, mature virions are released from the host cell through cell lysis, allowing the virus to infect new cells and continue the cycle. Interestingly, recent research suggests an alternative exit pathway via non-lytic mechanisms, such as exosomes [26]. However, the precise regulatory mechanisms governing this alternative pathway in relation to the lytic cycle remain unclear.

## Foot-and-mouth disease virus pathogenesis in vivo

### Disease stages and definitions

Similar to all infectious diseases, FMDV infection can be divided into distinct stages based on the extent of dissemination of the virus within the host, as well as the ability of the infected host to transmit infection to a different host. While the latent period is defined as the time elapsed from the moment of infection, until virus replication and shedding has reached levels required for transmission of disease, the incubation period represents the time from infection until the first clinical signs of disease appear. However, FMD can also manifest as a fully subclinical infection, referred to as neoteric subclinical infection, in animals with acquired immunity or genetic resistance to clinical FMD. The latent period is typically shorter than the incubation phase, meaning that transmission of infection can occur before development of apparent signs of disease [27–29]. Through the earliest phases of infection, viral replication occurs at primary sites, typically within the upper respiratory tract (nasopharynx) of ruminants and upper gastrointestinal tract (oropharynx) of pigs. Primary infection spans the latent phase and most of the incubation phase, before the virus disseminates systemically to reach secondary sites of replication (vesicular lesions). The clinical signs of FMD (defined primarily by fever and the presence of vesicular or ruptured lesions) typically resolve within 7–10 days after appearance, whereas the duration of the infectious period is more difficult to quantify experimentally. A single study in pigs showed that group-housed pigs were capable of transmitting FMD for at least 9 days [30], which is similar to an earlier experiment based on pair-housed cattle [31]. Vesicular epithelium contains very high quantities of infectious virus, which implies that any animal with residual vesicles should be considered contagious. This was further illustrated by isolation of infectious virus from vesicle epithelium of pig carcasses that had been stored at 4 °C for 11 weeks [30]. In ruminant hosts, FMDV may persist at a low level of replication within distinct epithelial tissue compartments in either the upper respiratory tract (cattle) or the upper gastrointestinal tract (sheep and African buffalo), a condition commonly referred to as the FMDV carrier state [6, 32–36].

### Incubation phase: primary infection

**Figure 2:**
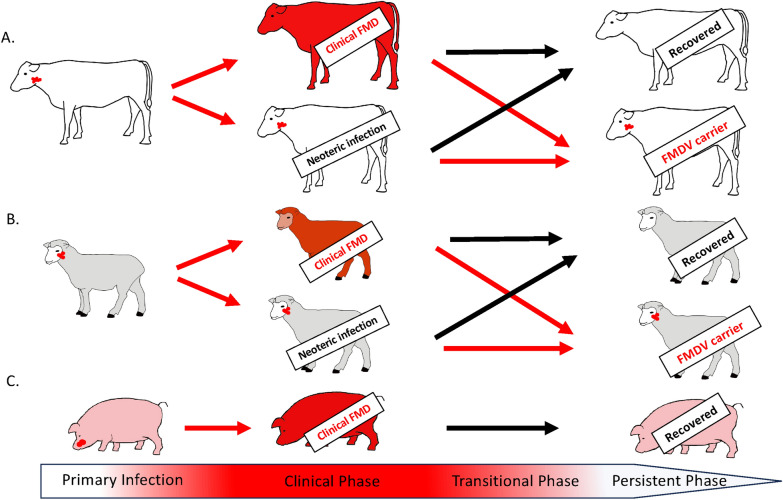
**Schematic illustration of FMDV progression in different domestic livestock species**. **A** In cattle, primary FMDV infection is localized to the nasopharynx (upper respiratory tract). Clinically susceptible cattle will progress through a phase of viremia and clinical disease, whereas animals protected by vaccination, previous exposure, or with breed-related resistance to clinical FMD will progress through a phase of neoteric infection during which infection is restricted to the nasopharynx, but virus shedding in oronasal secretions is common. Both clinically susceptible and protected cattle progress to either fully clear infection, or to maintain subclinical persistent FMDV infection of the upper respiratory tract through prolonged durations (the FMDV carrier state). **B** Sheep are infected via the respiratory tract (nasopharynx). The clinical severity of FMD varies greatly in sheep, and subclinical neoteric infections are common. Similar to cattle, sheep progress to either clear infection or become FMDV carriers. The site of virus persistence in sheep is localized to epithelial crypts of the oropharyngeal tonsils. **C** Pigs will primarily become infected via oral exposure to FMDV, with primary infection in the oropharynx, although the minimum infectious dose is higher than for cattle and sheep. Clinical signs of FMD in pigs are often dramatic, and infected animals shed very large quantities of virus. Pigs efficiently clear FMDV from all tissues following recovery from the clinical phase, and there is no FMDV carrier state in pigs.

The anatomic site of primary FMDV infection varies depending on host species and may also vary depending on the route of exposure (Figure 2). However, a common morphological epithelial tropism to reticular, or lymphoid-associated, epithelium has been demonstrated in distinct tissues of cattle, pigs, and sheep [37–42]. In cattle, the site of primary infection has been localized to the nasopharyngeal mucosa [38, 42], a feature that is consistent with the demonstrated sensitivity of cattle to infection via inhalation of FMDV aerosols [42, 43]. Shortly following infection of the surface epithelial cells, viral RNA and antigen can be detected within submucosal lymphoid follicles [37, 44]. Although the tonsils of the bovine upper respiratory- and gastrointestinal tracts do not function as sites of primary or persistent infection, the epithelial compartments of these tissues support substantial viral replication during the clinical phase of FMD [42, 44].

In contrast to cattle, pigs are highly resistant to infection via inhalation of FMDV [45]. Consistent with this, epithelial crypts of the oropharyngeal tonsils (the tonsil of the soft palate and the paraepiglottic tonsils) of the upper gastrointestinal tract have been identified as sites of FMDV replication during the very early stages of experimental FMDV infection in this species [39]. Similar to cattle, the epithelial components of the tonsil crypts also support FMDV replication throughout the clinical phase of disease [39]. Although detailed temporo-anatomic investigations of FMDV in wild boar are lacking, experimental investigations have shown a similar sensitivity to oropharyngeal (tonsil) inoculation in this species as in domestic pigs [46], suggesting a similar tissue-level tropism.

There are few detailed studies of early FMDV pathogenesis in sheep available. It has been shown that sheep are highly sensitive to FMDV infection via exposure of the upper respiratory tract through both direct deposition of inoculum, or through inhalation of aerosolized FMDV [40, 47, 48]. However, to our knowledge, there are no published records of the anatomic site of pre-viremic FMDV replication in sheep, and studies based on tissues harvested during the early stages of viremia identify both the nasopharyngeal mucosa and oropharyngeal tonsil crypts as potential sites or primary infection [40, 41]. In both cattle and sheep, inhalation of experimentally produced FMDV aerosols has been associated with substantial FMDV replication in the lungs during early stages of infection [41, 42]. These findings demonstrate that epithelial cells in the lower respiratory tract are permissive to FMDV infection when exposed to large quantities of virus. However, the feature of substantial amplification of FMDV in pulmonary tissues during early (pre-viremic) infection has not been confirmed using other modalities of virus exposure, including direct contact-exposure, and may thus be an artifact of the aerosol inoculation system used in the associated studies.

Asian water buffalo (*Bubalus bubalis*) is a common livestock species used for both dairy and meat production in many FMD endemic regions. Although detailed studies of the anatomic site of early FMDV infection in Asian buffalo are lacking, it may be reasonable to assume a high degree of similarity to cattle based on similar shedding and transmission patterns [49, 50]. African cape buffalo (*Syncerus caffer*) are considered a wildlife reservoir for FMDV. Detailed temporo-anatomic investigations of FMDV pathogenesis in this species are also lacking. However, a recent experimental study concluded that efficient within-group transmission of FMDV occurred despite lack of detection of FMDV in exhaled air, and with negligeable quantities of virus detected in nasal swabs [51]. Those findings suggest that airborne transmission of FMDV may be of less significance in this species compared to transfer of virus resulting from direct physical interactions between animals, and that, similar to pigs, the site of primary infection may not be located in the upper respiratory tract.

### The clinical phase of FMD

**Figure 3:**
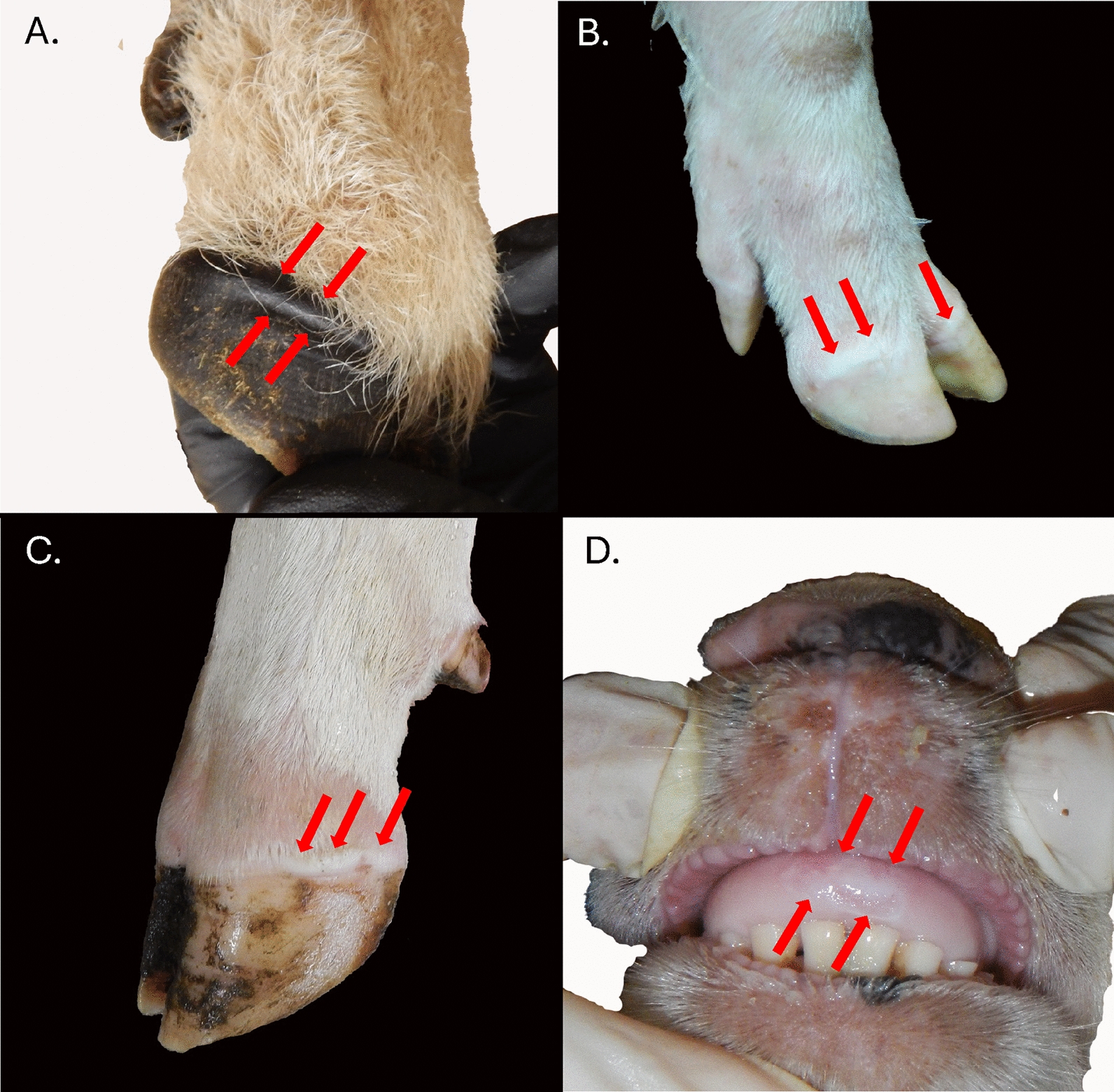
**Early stage FMD lesions in different host’s species**. Early FMD lesions are characterized by epithelial blanching and subtle vesiculation. **A** In sheep, coronary band vesicles often form beneath the upper portion of the hoof wall. Such lesions may be difficult to detect, and are typically associated with marked lameness and increased temperature of the hoof. Early FMD vesicles on the coronary bands of a **B** pig, and a **C** cow, characterized by blanching (white) skin and swelling. **D** Blanched and ruptured epithelium on the dental pad of a sheep, which is highly transient and resolves quickly.

**Figure 4:**
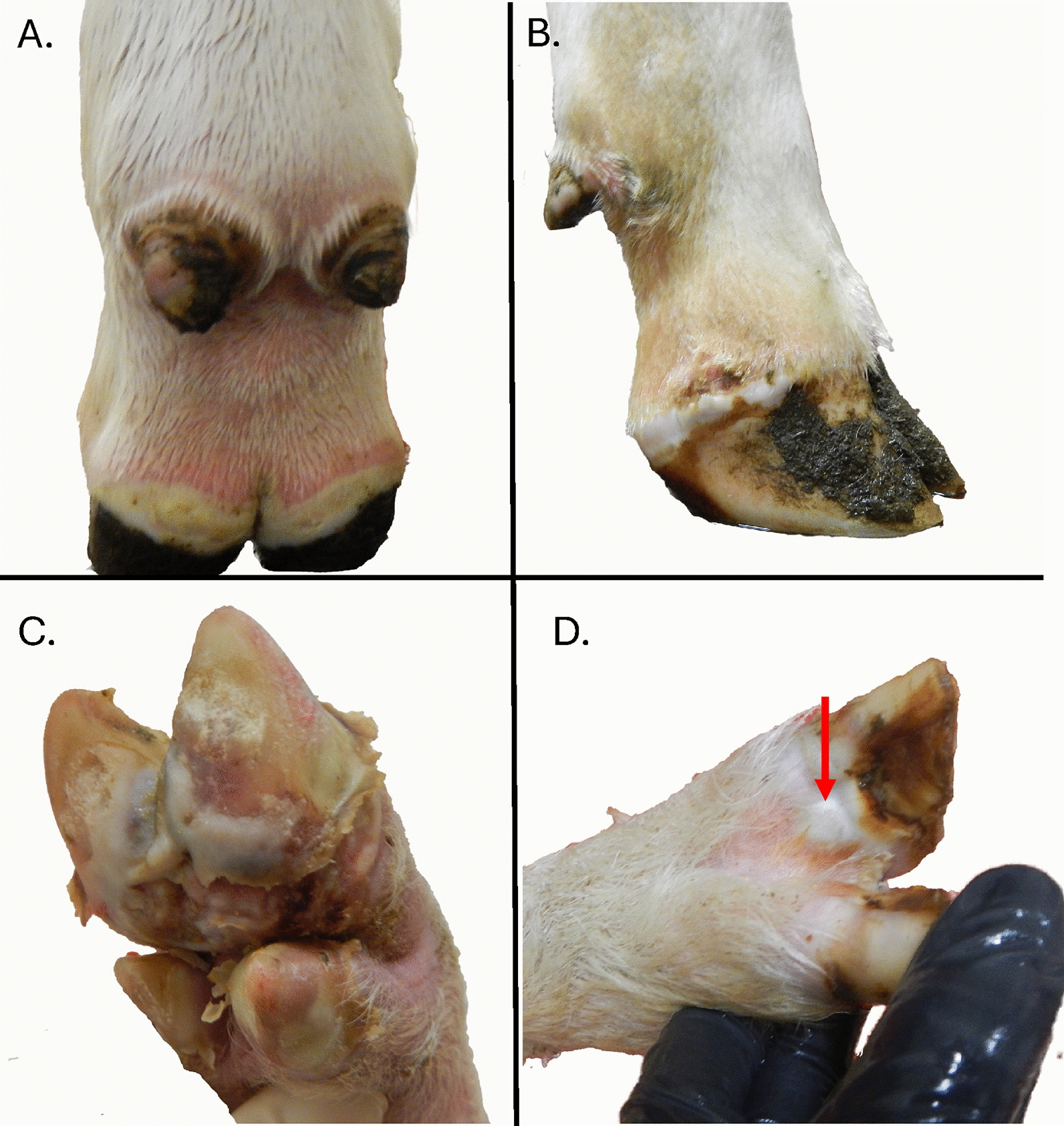
**Advanced FMD lesions in different host species**. Advanced vesicular lesions on the feet of FMDV infected **A**, **B**, cattle, **C** pig, and **D** sheep. Late-stage lesions often involve visibly thickened white to yellow epithelium that easily ruptures. Secondary bacterial infections of lesions are common.

The hallmark feature of clinical FMD is the appearance of characteristic fluid-filled vesicles in areas of non-haired skin, often concurrent with fever and viremia (Figures 3 and 4). However, although FMD lesions can be quite dramatic in naïve domestic cattle and pigs, the clinical signs of FMD can be far more subtle in animals that have some degree of pre-existing immunity, as well as in breeds or strains of animals that are naturally more resistant to the clinical manifestations of FMDV infection [52]. Even when vesicular lesions are present, the associated signs of pain and discomfort such as lameness and unwillingness to eat, stand, or walk may vary between host species. This phenomenon has specifically been reported based on experimental studies performed in wild-life species, including Eurasian wild boar, North American feral pigs, and North American bison (*Bison bison*) [46, 53, 54].

The conventional wisdom that sheep are asymptomatic for FMD or uniformly have mild symptoms is largely misguided. That said, experimental studies in sheep indicate that there is greater individual-animal variation in the clinical signs of FMD in this species, even amongst animals of similar genetic background and exposure history [55]. Sheep get gingival (mouth) lesions, but ptyalism (hypersalivation) does typically not occur. Also, as foot lesions and lameness due to other etiologies, especially bacterial infections, are common, the clinical signs of FMD in sheep may not be immediately noticed or appropriately recognized even when present. Unapparent FMDV infection in sheep was implicated as a major factor contributing to early dissemination of the UK 2001 FMD epidemic. However, reports from one of the earliest affected holdings document that several sheep were treated for lameness before being sent to market [56]. Thus, FMD in sheep is more likely to be overlooked or misdiagnosed due to the different, and often less dramatic, clinical presentation compared to cattle and pigs. Similar to sheep, studies in goats have reported subtle or absent clinical signs and substantial within-group variation in clinical presentations [57, 58]. Clinical FMD in Asian buffalo is often described as relatively mild [50, 59], although experimental studies based on direct inoculation of naïve buffalo have shown a sensitivity similar to that of cattle [49]. African buffalo are known to be largely resistant to any clinical manifestations of FMDV infection. However, recent studies showed that experimental infections in this species were associated with fever and subtle mouth lesions in some individuals [51].

Vaccination may prevent or mitigate the clinical features of FMD. However, the degree of protection will depend on multiple factors, including the homology of the vaccine virus and exposure strain, as well as the quality of the vaccine preparation and the timing of vaccination in relation to exposure. Vaccination typically does not prevent subclinical infection (i.e. not sterile protection), and although the level of viral shedding is generally reduced by vaccination, transmission may still occur. This occurrence of temporally acute subclinical infection, which can be distinguished from persistent FMDV infection based on virus shedding in oronasal secretions, is one form of neoteric subclinical infection. Neoteric infection also occurs when viruses and hosts are co-adapted, such as SAT strains in African buffalo or other endemic strains in indigenous cattle. In such instances, the clinical phase is replaced by a phase of similar viral shedding and/or viremia that is asymptomatic [51]. This phenomenon is specifically important as subclinical FMDV is usually overlooked in endemic settings where FMD surveillance and sampling are focused on clinical cases. An experimental study involving re-occurring sampling of repeatedly vaccinated dairy buffalo herds in Pakistan demonstrated repeated introductions and circulation of diverse strains of FMDV throughout the study period, in complete absence of any reports of clinical FMD in the herds [60]. That field study provides a relevant example of the extent to which FMDV may circulate amongst partially immune animals, without being noticed.

### Persistent FMDV infection in different host species

FMDV may cause a prolonged persistent phase of subclinical infection characterized by continuous low levels of virus replication within distinct micro-anatomic compartments in ruminant host species [32–34, 38, 61], but not in pigs [62]. The common definition of the FMDV carrier state involves isolation of FMDV from oropharyngeal fluid (probang) samples for more than 28 days after infection [63]. However, this universally accepted 28-day definition of FMDV persistence was derived from a single historical experimental study limited to 4 weeks duration, with the assumption that cattle that were virus-positive at the end of the study period were considered carriers [64]. The same publication reported that the cattle that successfully cleared infection, without developing persistent infection, had done so considerably earlier, a finding that has been confirmed by more recent publications [61, 65]. Thus, the “28 day threshold” of the FMDV carrier state holds no specific biological relevance, but may still be useful for practical purposes. Between the acute, neoteric or clinical phase of infection and the settled phase of subclinical virus persistence lies a transitional stage, during which some animals manage to fully clear FMDV from their tissues, while others fail to do so and go on to become carriers of the virus. The transitional phase has been demonstrated to occur at approximately 14–21 days post-infection (dpi) in clinically affected cattle, and approximately 7 days earlier (7–14 dpi) in cattle that were clinically protected by vaccination [38, 61, 65]. Although, the specific mechanisms of this transition remain incompletely elucidated, recent studies have suggested that establishment of the FMDV carrier state may be associated with impaired cell-mediated immunity and recruitment of phagocytic cells [66, 67]

The FMDV carrier state is both problematic and controversial, as infectious FMDV can be recovered from affected animals, but the levels of actual viral shedding and associated transmission seem to be minimal on an individual animal level [68]. However, the reported prevalence of persistent FMDV infection in cattle is about 50–100% at 4 weeks post infection, with a gradual decline through subsequent months to years [68–70]. Thus, given the very high prevalence of FMDV infection in endemic regions, the number of persistently infected animals present in the population at any given time can be incredibly high, especially in areas of recent FMD outbreaks. Even though the probability of transmission from any one FMDV carrier can be presumed to be very low, the high numbers of carriers present in the population may compensate for a low-probability event. Multiple small-scale experimental studies have failed to demonstrate transmission from FMDV carrier cattle [71–73]. However, circumstantial historical evidence, as well as limited experimental data suggest that transmission from carriers may occur [68, 74]. Based on this, it is difficult to conclude with certainty that there is no risk associated with the FMDV carrier state. Additionally, recent experimental studies have shown frequent events of viral recombination resulting in dominant inter-serotypic recombinant viruses in the upper respiratory tract of carrier cattle that are super-infected with a heterologous strain of FMDV [75]. Such animals are, in contrast to conventional carriers, simultaneously persistently- and neoterically infected, and may shed virus in oronasal secretions in absence of clinical signs of FMD.

In cattle, persistent FMDV infection has been localized to distinct regions of nasopharyngeal (upper respiratory tract) epithelium, similar to primary infection [38, 61]. In those regions, scant quantities of individual, or small clusters of epithelial cells remain infected with no evidence of an activated immune response or the lytic properties that accompany acute infection [38, 61]. Transcriptomic studies have indicated that maintenance of persistent FMDV infection is associated with impairment of apoptotic pathways and Th17 mediated immune responses [76, 77]. Overall, these studies suggest that the carrier state is associated with dysregulated regional (mucosal) immune function, however, confirmation of immune pathways involved in both establishment and maintenance of the FMDV carrier state remains incomplete. One investigation has reported detection of FMDV RNA and structural antigen in lymph nodes draining the oral cavity [35], however, there are no reports of recovery of infectious virus from any other sites than the nasopharyngeal mucosa. Segments of the nasopharyngeal mucosa can be accessed through probang sampling, which involves inserting a specifically designed metal cup into the oral cavity of the animal. Insertion of the tool induces a swallowing reflex, during which the soft palate is elevated to enable scraping of the mucosal surface of the dorsal nasopharynx and the caudal aspect of the dorsal surface of the soft palate [6, 78]. For successful recovery of FMDV from the oropharyngeal fluid (OPF) samples obtained by probang sampling, the sample should contain some cellular debris, without contamination by rumen contents.

The FMDV carrier state in small ruminants is less well-studied compared to cattle, and has been demonstrated to have both similarities and differences. In sheep, there is an important anatomic distinction from cattle as persistent FMDV has been localized primarily to epithelial crypts of the oropharyngeal tonsils (palatine- and paraepiglottic tonsils) rather than to the nasopharyngeal mucosa [34, 41]. Burrows described a modified approach to probang sampling of sheep, which results in mucous scrapings rather than a liquid sample [34]. Despite the different location of persistent infection in sheep compared to cattle, the probang is still being passed across the oropharyngeal openings of the palatine tonsil crypts during the sampling procedure. In our experience, the frequency of successful virus detection in probang samples from sheep increases when specifically targeting the area of the palatine tonsil crypts (dorso-lateral oropharynx) rather than the more caudal sampling approach used for cattle. There is limited information available regarding the duration of persistent FMDV infection in sheep. In a study involving 35 animals, Burrows reported that 80% of animals were confirmed carriers at 4 weeks after infection, 25% at 12 weeks, and a single animal was still positive at 20 weeks [34]. A purported shorter duration of FMDV persistence in small ruminants could contribute to less frequent detection of carriers among these species during field surveys [58].

Experimental studies in African buffalo have shown that although FMDV can be detected in probang samples from persistently infected animals, the frequency of virus detection was greater when using cytology swabs to access the palatine tonsil crypts on sedated animals [36]. This finding suggests that the anatomic site of persistent FMDV infection in African buffalo may, similar to sheep, be primarily localized to the epithelial crypts of the palatine tonsils. Interestingly, multiple detailed studies of persistent FMDV in cattle have failed to detect any FMDV in the palatine tonsils, while consistently detecting virus in samples of the nasopharyngeal mucosa [38, 61, 79]. These findings suggest that although persistent FMDV infection is a common feature in ruminant host species, the macro-anatomic site of persistent infection is not similar across species, whereas micro-anatomic and mechanistic similarities may be preserved.

### Viral determinants of FMDV persistence

The potential contributions of specific viral genetic determinants to FMDV persistence have been investigated in both field and laboratory settings [71, 80–83]. Although some publications have reported significant findings, including specific amino acid substitutions in VP2 [83] or VP1 [82], there is lack of consistency across publications. Maree et al., reported competitive advantage and greater prevalence and duration of persistence of a SAT-1 virus, versus SAT-2, and −3 in African buffalo that had been simultaneously coinfected with all three viruses [36]. That finding suggests that certain FMDV strains may be more likely to cause persistent infection than others. By contrast, experimental studies based on simultaneous or sequential coinfection of cattle with FMDV strains O1 Manisa and A24 Cruzeiro found no relative advantage of either virus [75, 84]. Overall, the mechanistic determinants of the carrier state are still incompletely understood, and current evidence suggests that both host and viral factors may be involved.

### Atypical clinical manifestations of FMD: myocarditis and abortion

**Figure 5:**
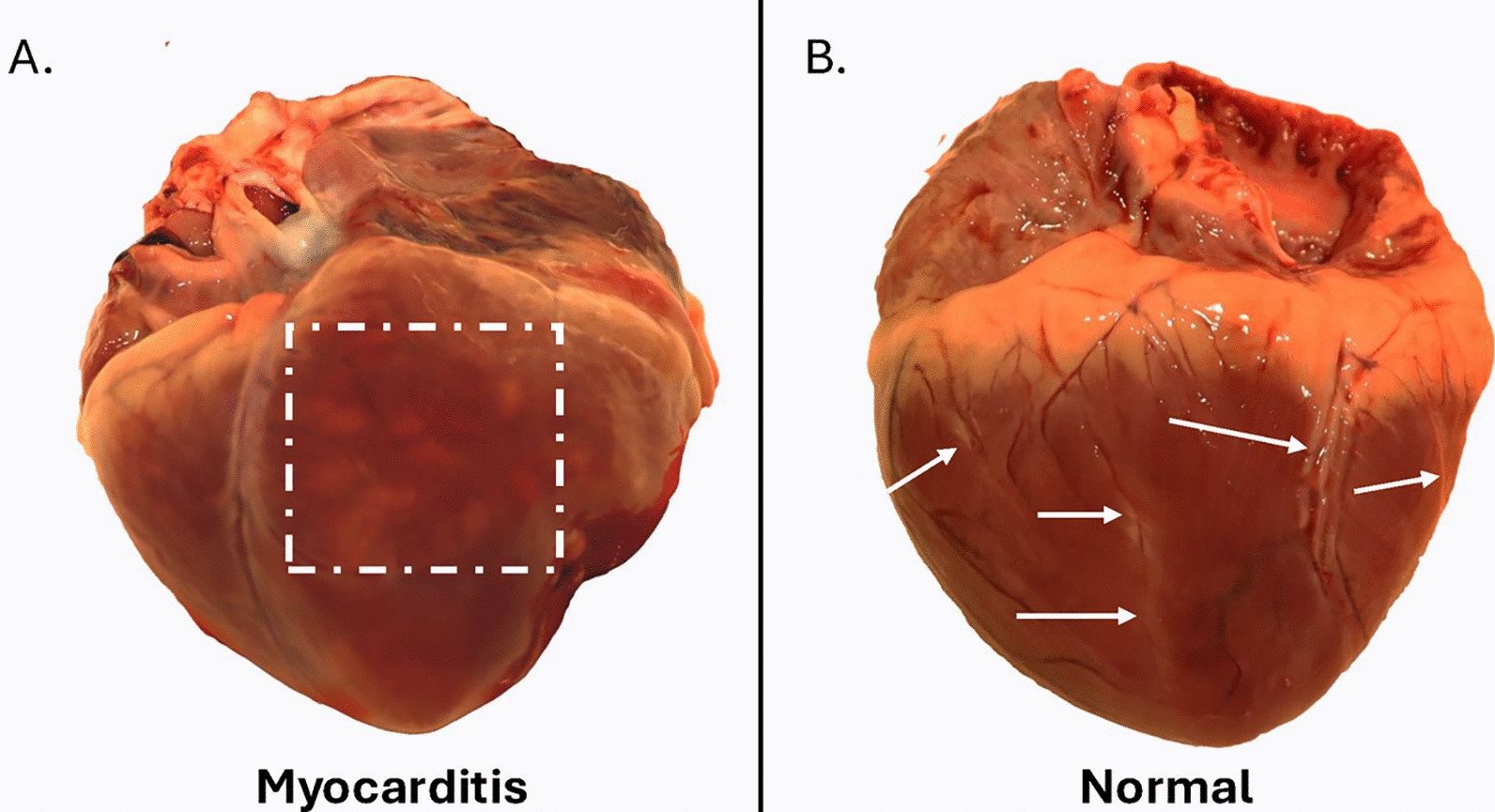
**Porcine hearts with and without FMDV-associated myocarditis**. **A**. Heart from pig that died of FMDV-associated myocarditis at 4 days post experimental infection with FMDV A24 Cruzeiro. Within the region of interest marked with a hatched square, multifocal well-demarcated areas of pallor are present on the epicardial surface of the left ventricle and through the myocardium. This lesion morphology is typical for FMD myocarditis cases, and does not resemble stripes. **B**. Normal heart of pig without gross lesions of FMD myocarditis. Several normal anatomical structures of the myocardial surface, including vasculature tracts and epicardial fat (white arrows) resemble stripes. These normal structures indicate why the commonly used term “tiger heart” is inappropriate to describe FMD myocarditis as it encourages misdiagnosis of normal features of the myocardium as potential lesions. The image in panel A has been edited to remove glare from lights, without altering other features of the image.

FMD is a known cause of fatal myocarditis, particularly affecting juvenile animals [4, 85]. However, the incidence of FMD-associated myocarditis varies depending on both viral and host factors, and these underlying factors are incompletely elucidated. FMD-associated myocarditis has historically been described as “tiger heart”. This terminology is an unfortunate misnomer as it does not accurately describe the typical gross pathological lesions in animals that have succumbed to FMD-associated myocarditis. Further, some normal anatomical structures of the heart, including epicardial vasculature and fat, have a stripe-like appearance, and can easily be misinterpreted as features resembling a “tiger heart” by inexperienced examiners. The typical progression of FMD-associated myocarditis is rapid, and death may occur with neither clinical manifestations of FMD nor congestive heart failure. In rapidly progressing cases, gross lesions of the myocardium may be absent. When gross lesions are present, these occur as areas of pallor (pale color) of the myocardial surface (Figure 5) that extend into the myocardium when the surface is cut [86]. Histologically, mononuclear infiltrations and edema are seen combined with cardiomyocyte necrosis [87, 88]. Immunohistological staining for FMDV will reveal high quantities of viral protein in the myocardium [88]. As FMD-associated myocarditis occurs concurrent with viremia, detection of FMDV in myocardial samples by PCR or virus isolation is not sufficient for diagnosis of myocarditis, which requires identification of gross and/or histological lesions.

FMD has also been associated with fetal death and abortion in both sheep and cattle [89, 90]. Limited experimental and field-based reports similarly identified fetal infection with or without death or abortion. However, these events are sporadic rather than consistent, and likely depend on the timing of virus exposure during gestation, and possibly also on unknown viral strain and host genetic factors.

## FMDV transmission

FMD is a highly contagious disease, and the most apparent link of that contagiousness to pathogenesis are the large quantities of FMDV that are shed during infection and the diverse routes and mechanisms through which animals may become infected. Over recent decades, research efforts have substantially advanced the understanding of FMDV transmission dynamics. Early disease control strategies often rely upon rapid detection of clinical signs to initiate and guide interventions. However, it is now well established that FMDV transmission can occur before clinical signs are apparent [28, 29, 56], posing substantial challenges for surveillance and response. Moreover, various aspects of FMDV transmission complicate control including environmental and fomite contamination, feed-mediated transmission, neoteric infections, persistent infections, wildlife reservoirs, and variable vaccine performance. Through progressively refined experimental designs, mathematical and Bayesian modeling approaches, threshold-based measures of infectivity, and genetic analyses, researchers have gained improved insights into critical parameters such as the minimum infectious dose (MID), the proportion of preclinical transmission, and the epidemiological significance of persistent carriers and wildlife. Significant knowledge gaps remain despite these advances having enhanced outbreak models and informed better-targeted interventions. Efforts to standardize experimental designs, integrate molecular and epidemiological tools, and expand research to diverse ecological settings will further strengthen FMD control globally.

### Preclinical transmission dynamics

The concept that infected animals can shed FMDV and cause new infections before the onset of clinical signs has important implications spanning pathogenesis, mathematical modeling, surveillance, and control. Initial recognition of preclinical (incubation-phase) transmission came from studies by Orsel et al. [29, 91], who introduced the “non-clinical” reproduction ratio (R_nonclin). These studies revealed that certain host species, particularly pigs and dairy cows, could drive considerable silent spread. Subsequent identification of the nasopharynx as the primary site of FMDV replication following aerosol exposure in cattle, provided additional insights into early infection dynamics and demonstrating the potential for preclinical shedding to contribute to transmissional [42]. Charleston et al. [92] used Bayesian frameworks to estimate the proportion of preclinical transmission (θ), finding a θ of approximately 0.13 in cattle based on observed transmission events but much higher estimates (up to ~0.44) when relying on proxy measures of viral presence rather than confirmed secondary infections. Arzt et al. [27] similarly applied Bayesian models in a prospective experimental study to examine preclinical transmission in pigs, clearly inculpating the existence and relevance of preclinical transmission.

Experimental designs incorporating sequential exposure and threshold-based measures have enhanced our understanding of preclinical shedding. Stenfeldt et al. [28] pinpointed the onset of infectiousness in pigs about 24–32 hours post-infection (hpi), well before clinical signs. Eblé et al. [93] demonstrated that vaccination, when timed appropriately, reduced viral shedding and transmission among pigs, showing a potential to mitigate preclinical spread during outbreaks. Accelerated Failure Time models and threshold-defined shedding measures were used to refine estimates of infection phase durations in cattle and pigs [94, 95]. Integrating these findings, Gubbins [96] incorporated within-host viral load kinetics into models, allowing for precise serotype- and route-specific predictions of preclinical spread.

Collectively, these studies emphasize that preclinical transmission is neither rare nor negligible. Accounting for preclinical spread is essential for accurate disease models, effective early detection, and timely interventions. The growing use of threshold-based metrics and Bayesian inference [27] exemplifies how refined methodologies can yield more reliable parameter estimates, ultimately improving outbreak prediction and control strategies.

### Persistent infection and carriers

The epidemiological relevance of persistently infected “carrier” animals remains an area of intense debate. Arzt et al. [97] demonstrated that unadulterated oropharyngeal fluid (OPF) from carrier cattle could induce clinical disease when deposited into the upper respiratory tract of naïve recipients. However, field evidence for carrier-driven transmission is minimal. Bertram et al. [98] and Parthiban et al. [71] found no carrier-to-susceptible transmission under natural husbandry conditions, and a meta-analysis of carrier transmission studies identified only a few successful carrier-mediated transmissions, primarily from African buffalo (*Syncerus caffer*) to cattle or other buffalo [68].

This species-specific discrepancy is crucial. African buffalo are important reservoirs for Southern African Territories (SAT) serotypes [99], maintaining long-term infection cycles. Comparatively, carrier cattle in field conditions appear to pose a negligible risk. Nevertheless, the carrier state should not be dismissed. Arzt and Stenfeldt et al. [75, 100] have documented that heterologous FMDV superinfections in carrier cattle often lead to viral recombination and shedding of multiple strains, raising concerns about their potential role in FMDV evolution. Further, experiments in which cattle were simultaneously co-infected with the same virus strains did not result in any recombination events [75]. Thus, super-infection of FMDV carrier cattle is at this time the only documented in vivo source of recombinant FMDV strains.

### Indirect and environmental transmission

Complex pathways beyond direct contact significantly influence FMDV epidemiology. Studies on aerosolized viruses [101] and contaminated environments [102] have shown that naive calves can become infected under controlled conditions, even without direct contact with infected animals. Contaminated rooms and surfaces can maintain infectious virus that may initiate infection in susceptible animals.

The transmission dynamics of FMDV among different species have been explored, highlighting species-specific variations in susceptibility and transmission efficiency. Fukai et al. [103] investigated horizontal transmission using FMDV strain O/JPN/2010 and found that while within-species transmission occurred consistently, cross-species transmission between pigs, cows, and goats was not uniformly successful. For instance, pig-to-cow transmission was observed, but cow-to-goat or goat-to-cow transmission was not detected, underscoring the influence of host-specific factors on transmission potential. Studies of O and A viruses isolated during the 2010 outbreaks in the Republic of Korea demonstrated that strain-specific differences may also affect species specific transmission dynamics [104]. These findings emphasize the importance of tailoring control strategies to address species-specific risks and interactions.

Feeding pigs contaminated waste or commercial products has been implicated as the route of incursion of FMDVs [105]. Experimental studies have demonstrated how feed-mediated transmission is affected by exposure frequency and duration, and host susceptibility shape outbreak dynamics. Stenfeldt et al. [106] found that multiple high-dose exposures to contaminated feed were necessary to infect pigs. Similarly, differences in susceptibility to aerosol routes have been documented. For example, Van Roermund et al. [9] explored between-pen transmission in pigs, finding that although solid barriers prevented between-pen transmission from vaccinated pigs, limited transmission still occurred from non-vaccinated seeder pigs, suggesting that short-distance aerosols or minor indirect contacts facilitated infection. Other investigations have concluded that pigs are largely resistant to FMDV infection via inhalation [10, 45], whereas cattle and sheep are susceptible to low doses of aerogenous FMDV [43, 107, 108]. Such differences have implications for practical aspects of control and risk assessments. Understanding dose–response relationships [109] and threshold-defined infectivity can help pinpoint conditions under which indirect routes drive outbreaks.

### Wildlife reservoirs and the livestock interface

Wildlife species can maintain and spread FMDV, often complicating eradication efforts [110]. African buffalo are well-established reservoirs for SAT serotypes, sustaining transmission cycles in mixed wildlife-livestock systems [68, 99]. Genetic analyses by Blignaut et al. [111] have revealed overlapping viral lineages in wildlife and livestock, confirming ongoing cross-species exchange. Surveillance by Alexandrov et al. [112] demonstrated that while wild boar and deer can become infected and may cluster seropositive individuals during outbreaks, they likely cannot maintain FMDV at the population level without livestock involvement. This finding suggests that in FMD-free regions, wildlife may serve as bridge species during outbreaks of infected livestock but may not serve as independent reservoirs in all settings. An historical exception occurred during the 1924-25 outbreaks in California, in which 22 000 mule deer were culled, due to pervasive infection [110].

Environmental factors like shared contaminated water or feed resources can further facilitate cross-species transmission [113]. These complexities highlight the need for more refined methods to quantify wildlife-livestock contact rates, ensure improved surveillance of wildlife populations, and integrate ecological context into disease transmission models.

### Modeling transmission dynamics and the minimum infectious dose

Mathematical modeling has been instrumental in quantifying FMDV transmission parameters, including phase durations and spread rates. Early models [114–116] calculated basic reproduction numbers (R_0) in unvaccinated, within-pen pigs as high as 30–40. Dekker et al. [117] revisited R_0 estimates in pigs, finding consistent results for unvaccinated pigs of 39 (95% CI 29–59) while also demonstrating elevated spread rates of 3.7 (95% CI 1.9–7.3) in vaccinated groups. Gunasekera et al. [118] employed six analytical methods, including epidemic doubling time, stochastic doubling time, nearest neighbor infection, time-dependent reproductive number (TDR), sequential Bayesian (SB), and BEAST2 Birth–Death Skyline (BDSKY) analysis, to estimate the between-population reproductive number (Rbp) in Vietnam. Across these approaches, Rbp values consistently ranged from 1.25 to 1.61. Despite methodological differences, the results were comparable, with minimal variation attributable to the analytical method.

Integrating preclinical infectiousness into models [27, 29] and applying threshold-defined infectivity estimates [94, 95] have refined transmission estimates.

Orsel et al. [29] found a low risk of non-clinical transmission (R_nonclin) in calves and lambs, but R_nonclin > 1 for dairy cows and pigs. Accelerated Failure Time (AFT) models have been applied to compiled data from experimental FMDV studies to account for virus, host, and methodological influences in phase duration in both cattle and pigs [94, 95]. The mean incubation period in was estimated pigs to range from 2.6 to 3.2 days (mean 2.9 days), with minimal differences across virus strains or exposure methods [95]. By comparison, a longer mean incubation period, at 3.6 days (95% CI 2.7–4.8), was estimated for cattle, with considerable serotype and exposure method variability [94]. The preclinical infectious period in pigs was estimated to an average of 1.5 days, influenced by experimental variables such as virus strain and exposure method [95], whereas a longer subclinical infectious period was reported for cattle, averaging 2.2 days (95% CI 1.5–3.5), similarly noting that serotype and exposure method played significant roles [94].

Estimates of the preclinical transmission (θ) proportion for FMDV vary substantially across studies due to methodological differences, species-specific factors, and reliance on proxies for infectiousness. Arzt et al. [27] estimated θ at 0.12 (95% CI 0.00083–0.27) for pigs, indicating that 12% of FMDV transmission occurs during the subclinical phase. This estimate, derived from Bayesian modeling, emphasizes the low but meaningful contribution of preclinical transmission in pigs. By comparison, Charleston et al. [92] estimated θ to be 0.13 in cattle using direct transmission measures but reported substantially higher θ values (e.g., 0.43, 0.27, and 0.44) when using proxy measures such as viral isolation from blood, nasal fluid (NF), or oropharyngeal fluid (OPF). Using similar data, Gubbins et al. [96] estimated θ as 0.18 (95% CI 0.06–0.43) with nasal fluid as a proxy and higher estimates (0.32–0.45) when blood or OPF proxies were used.

Taken together, these findings highlight that while modeling efforts have greatly enhanced our understanding of FMDV spread, the resulting parameter estimates remain sensitive to the chosen methods and assumptions. Continued refinement of analytical frameworks, standardized experimental designs, and carefully selected proxies will help ensure more robust and comparable parameter estimates across epidemiological scenarios.

## Pathogenesis of the host response to FMDV

FMDV infection is highly cytolytic in individual cells, but it is rarely lethal to the host at large. Innate defenses come into play to restrict initial infection, to protect other cells from getting infected, or to eliminate already infected cells. Innate immunity acts without delay, but only serves to slow, rather than stop, the infection, allowing time for the slower antibody- and cell-mediated adaptive immune response to take over [119].

Each animal species responds differently to FMDV infection. Species-level tropism is not dependent on receptor availability alone, and examining variations in local and systemic immune responses—both innate and adaptive—can contribute to a better understanding of host-specific FMDV pathogenesis [120, 121].

### Host-virus interaction in the early phase of infection

At the sites of primary replication, the virus interacts with a complex network of host cell factors, cellular signaling pathways and specific proteins that can promote or inhibit viral replication at different stages (Table 1). Viral RNA and replicative intermediates are detected by nucleic-acid-sensing receptors in the endosome (TLR7/8, TLR3) and cytosol (RIG-I, MDA5), triggering a signaling cascade that ultimately leads to the induction of type I interferon (IFN) (through IRF3/7) and other proinflammatory cytokines (through NF-κB) such as TNF-α, IL-1 and IFN-γ [122]. Type I IFN can be produced by virtually all nucleated cell types [123]. In FMDV-infected cattle, plasmacytoid dendritic cells (pDCs) have been proposed as a major source of IFN [124], but it is unclear how much of the systemically detected IFN originates from within the vasculature rather than from infected tissues [125]. Either way, released type I IFN then binds specific receptors on other cells, resulting in the transcription of hundreds of so-called IFN-stimulated genes (ISGs) and inducing an antiviral state. Type I IFNs also activate natural killer (NK) cells and induce other cytokines such as interleukin (IL)−12 to promote NK responses. NK cells are an important component of the innate defense against viruses, produce proinflammatory cytokines, kill infected cells and interact with dendritic cells (DC) [119]. In pigs, however, NK cell responses are impaired during acute infection, coinciding with viremia, lymphopenia and fever [126]. Maturation of dendritic cells (DC) is promoted by type I IFN, influencing the efficacy of the adaptive immune responses induced. In the clinical phase of FMDV infection, DCs are co-opted by the virus to produce IL-10, which skews the immune response towards a humoral rather than a cell-mediated adaptive response [127].

During the long co-evolution with its hosts, FMDV has acquired many other strategies to actively counteract the host antiviral responses [123, 128], increasing its replication and promoting its onward spread (Table 1). This antagonism can be roughly divided into three categories: (1) interfere with the function of the innate immune response, (2) misdirect the cellular immune response, (3) escape the humoral immune response. For picornaviruses, the length of the genome is strongly constrained by the size of the capsid, putting pressure on the virus to economize protein function [129]. Accordingly, all viral proteins have a variety of roles in the viral life cycle (Table 1). While most of the subversion of the host immune response is mediated by non-structural proteins (primarily the proteases Lpro and 3 C), the capsid proteins also have immunomodulatory functions in addition to their main tasks of protecting the viral genome and binding cellular receptors: VP0, the cleavage precursor of VP4 and VP2, suppresses the activation of the IFN-β promoter by interacting with PCBP2 [130], while an interaction between FMDV VP2 and HSPB1 activates the EIF2S1-ATF4 pathway leading to increased autophagy [131]. Autophagy is an ancient and conserved biological process, which exists in almost all eukaryotes. While originally a defensive mechanism, many viruses have hijacked parts of the autophagy pathway to promote their own replication, and it is likely that FMDV does, too [132].

VP3 interacts with the cellular adaptor protein VISA, blocking the IFN-β signaling pathway [133]. Many of the immunomodulatory functions described herein target the type I IFN response, whereas the capsid protein VP3 was also shown to degrade JAK1 to inhibit phosphorylation and dimerization of STAT1, interfering with host cell responses to type II IFN-γ signaling [133].

VP1 interacts with sorcin to inhibit type I IFN and disrupt the signal transduction of NF-κB [134], promotes the degradation of TLP2 to reduce the expression of antiviral cytokines [135] and enhances MAPK signaling by counteracting the host ribosomal protein SA. Apart from promoting FMDV replication, the unbalanced activation of the MAPK pathway might be directly pathogenic by driving the excessive inflammatory reaction in the oral cavity and the coronary bands observed during FMDV infection [136].

Conceptually, the degradation of the host immune response and the promotion of viral replication are distinct viral functions, but they are often achieved by the same mechanism. This is probably best demonstrated by the phenomenon known as “host shut-off”. The primary eukaryotic translation initiation pathway depends on recognition of the 5′ cap on an mRNA. An internal ribosome entry site (IRES) is a cis-acting RNA element that allows ribosomes to bind and initiate translation without cap recognition, which allows protein synthesis to continue when cap-dependent translation is inhibited, and many viruses including FMDV have evolved to make use of this [137]. FMDV Lpro (and 3 C) cleave the host translation initiation factor eIF4G, which is essential for cap binding [138], and 3 C cleaves histone H3 resulting in a generalized repression of cellular transcription [139]. Thus, as a direct result of FMDV infection, host cell protein synthesis is rapidly shut off without affecting translation of viral mRNA, diverting the cell protein synthesis machinery to the production of large amounts of virus and terminating the production of immune mediators.

Apart from its interference with host protein translation in general, Lpro specifically counteracts immune functions [140]. It exerts pressure on the type I IFN response by decreasing IRF-3/7 protein levels [138] as well as cleaving TBK1 and MAVS [141] and affects NF-κB activity by degrading p65/RelA [142]. Lpro and 3 A both suppress the stress response associated with viral infections by cleaving stress granule scaffold proteins [143] and degrading the stress granule assembly factor G3BP1 [144], respectively.

In an interesting example of synergistic action of two viral proteins with very different main functions, the viroporin 2B counteracts the inhibition of Lpro and 3 A by the porcine host protein CypA [145]. 2B itself also interferes with RLR-mediated type I IFN signaling [146, 147].

The non-structural protein 3 A is known to play important roles in FMDV infectivity, tropism, and replication. Together with 3B, it is a strong determinant of host range and virulence [148]. Viruses with large deletions in 3 A can no longer infect bovine cells nor cause disease in cattle, but retain virulence in swine [149]. Interestingly, the clinical attenuation in cattle does not appear to be caused by a difference in the host response, but by the reduced replication efficiency of the 3 A mutant virus [44]. At the same time, 3 A was shown to interact with RIG-I like virus-sensing receptors to inhibit the induction of IFN-β signaling [150] and with DDX56 to reduce phosphorylation of IRF3 [151].

Finally, the 3 C protease, which does most of the viral polyprotein processing in FMDV replication, also cleaves NEMO, an essential adaptor protein in the NF-κB and IRF signaling pathways, interrupting both the production and action of type I interferon [152]. It also interferes with the type II IFN signaling pathway by blocking STAT1 nuclear translocation [153].

While some cellular proteins are targeted by only a single viral protein, other targets are shared by several FMDV proteins. This is particularly notable for the RLR and NLR pathways responsible for sensing viral RNA and activating the innate immune response. The diversity of viral proteins responsible for the inhibition of the induction phase of the IFN pathway and the diversity of the targeted cellular proteins indicates the importance of this interference for the viral life cycle. At the same time, it allows a very fine regulation of the virus-host equilibrium [154].

Perhaps paradoxically, despite the multitude of ways in which FMDV counteracts the innate immune response, infection of cattle leads to a robust systemic activation of type I and III IFNs as well as acute-phase proteins [155]. By contrast, the systemic response in FMDV-infected pigs is more variable [121]. An immunosuppressive stage modulated by IL-10 during acute FMDV infection of this species has been reported [127, 156], with elevated IL-10 levels contributing to lymphopenia and potentially dampening antiviral activity. Similarly, studies in mice have suggested that FMDV-induced lymphopenia can be prevented by blocking IL-10/IL10R signaling, improving survival post-FMDV infection in this species [157]. However, whether the mechanisms demonstrated in mice translate to other, natural, host species of FMD is unclear. Additionally, the relevance of IL-10 and potential lymphopenia during FMDV infection in cattle is contested [59, 158–160] and should be investigated further, particularly with regard to its possible impact on the carrier/non-carrier divergence [161].

### Host-virus interaction in the late phase of infection

The rapid activation of B cells after FMDV infection leads to an early antibody response, as evidenced by detectable serum IgM within 3–4 days after infection in cattle, followed by peaks of IgA and then IgG within the following 1–2 weeks [162–164]. With the onset of the systemic neutralizing antibody response, viremia ceases and acute disease symptoms fade. In general, systemic antibody responses to FMDV infection are long-lasting: humoral protection against reinfection was demonstrated as late as four to 5 years after infection and may persist for the effective lifetime of most cattle [162]. This assumption, however, is based on only a very small number of studies and more data are needed.

As for the mucosal immune response following infection of cattle, a peak of neutralizing activity attributed to IgM and IgA was observed in OPF a week after virus exposure but this was attributed to a leakage of serum and tissue fluid into the oronasal cavity. Later, during the persistent phase of infection, neutralizing activity in OPF stems from local production of IgA rather than serum transudation [165] and the constant local immune stimulation leads to a strong FMDV-specific IgA response in nasal and oral secretions of persistently infected animals. By contrast, there is no difference in the systemic response between carriers and non-carriers. In particular, a persistent serum IgM response, which is pathognomonic for chronic active viral infections in humans, is absent, suggesting a lack of stimulation of the systemic immune response in FMDV carriers [166]. While the strong local IgA response may be useful for the diagnosis of persistent infection [61, 167], it does not lead to clearance of persistent virus from the nasopharynx. Animal-level variability in antibody secretion [61], and the need for a matching virus for antibody capture leave this approach suboptimal for diagnostic purposes. The failure to clear persistent infection is not due to a lack of neutralizing capacity of the IgA itself [166], but intracellular virus at the mucosal sites of persistence is inaccessible to antibody. There also is no accumulation of FMDV structural proteins in the lipid membrane of infected cells and thus no role for antibody in direct cytotoxicity mechanisms for the elimination of these cells [168]. The cell-mediated adaptive response, on the other hand, appears to be directly impaired in persistently infected tissues [66, 77].

The role of exosomes in both activation and evasion of FMDV-induced innate immune responses is still being investigated [26]. Exosomes are membrane-bound vesicles that act as intercellular messengers and may play a functional role in mediating immune responses in the face of infection [169]. On the other hand, some picornaviruses such as hepatitis A virus have co-evolved to use exosomes to spread undetected by humoral immunity [123] and it is possible that FMDV makes use of exosomes in a similar manner [170]. The improved recovery of infectious virus from OPF samples after treatment with trichlorotrifluoroethane (TTE) [171] that is usually attributed the dissociation of virus-antibody complexes [166] may also be at least partly due to a release of virus from intracellular compartments or exosomes.

### Response to vaccination

Vaccination can play a central role in the control of FMD outbreaks by reducing both the impact of clinical disease and the extent of virus transmission between susceptible animals. All currently commercially available FMD vaccines are inactivated whole-virus vaccines with oil-based adjuvants or, for use in cattle only, aluminum hydroxide and saponin [172]. Vaccination can be protective against clinical disease and virus shedding as early as 4–7 days after the first dose in cattle [173], sheep [174] and pigs [175].

Systemic antibody responses to immunization generally are of shorter duration and lower titer than in the response to natural infection [168], but field studies conducted several years after the end of annual vaccinations in Europe still found a high prevalence of presumably protective levels of neutralizing antibodies [176, 177]. Mucosal responses are more fleeting [166]. At the same time, only a strong mucosal response could conceivably prevent primary infection and subsequent virus persistence; accordingly, currently available vaccines generally fail to do so [178], unless very high antigen doses are used [179, 180].

### Effect of FMDV infection on the host transcriptome

Changes to the host transcriptome after FMDV infection are largely a result of the diverse interactions of the virus with the host immune system described above, including both direct viral effects as well as secondary inflammatory responses.

A diverse body of work deals with transcriptomic profiles of acutely or persistently infected permanent cell lines, e.g. BHK-21 [181–183] or PK-15 [184] cells. What is missing, however, is a comprehensive meta-analysis of these studies to identify common themes.

Gene expression studies on postmortem samples from persistently infected ruminants have been conducted using real-time RT-PCR and whole-transcriptome microarrays, using either macroscopically cut pieces of mixed tissue or specific tissues isolated by laser capture microdissection (LCM). These studies have examined both the transitional stage between one and 3 weeks after initial infection, during which cattle are in the process of clearing infection or transitioning to become persistently infected carriers, and the later stage of settled persistent infection. Many of these studies report the suppression of antiviral host factors and an anti-inflammatory state in tissues of carriers taken from the sites of persistent infection in the nasopharynx [61, 66, 79].

In the LCM study, the only study that included samples taken during the transitional phase, it was shown that clearance of FMDV from the follicle-associated epithelium of the nasopharyngeal mucosa was associated with the activation of the cytotoxic T cell response (Th1 polarization) as well as pro-apoptotic mechanisms [66]. Conversely, gene signatures associated with a reduced recruitment of neutrophils and cytotoxic T cells, a suppression of apoptosis and an impairment of cytotoxic T cell function (Th2 polarization) were observed in persistently infected carriers at later stages of infection both in the microdissection study and in other studies on macerated whole tissues [67, 76]. This may be indicative of a microenvironment created by the immunosuppressive effects of inducible Tr1 Treg cells [77]. Further investigations to elucidate immune mechanisms associated with the FMDV carrier state divergence should include a functional characterization of local FMDV-specific T cell responses during the transitional and persistent phase of infection.

During the early phase of FMDV infection, a strong antibody response is critical for removing the virus from the circulation and limiting acute disease. The preference for a strong Th2-mediated response may be a consequence of the positive selective pressure towards quickly clearing acute, systemic, infection, compared to preventing the strictly localized persistent infection, which in itself is not detrimental to the health of individual animals [66].

Microarrays have now been all but replaced by RNA-seq as a method for comprehensive unbiased transcriptomic analysis. So far, no published studies have applied RNA-seq to analyze ex vivo samples from persistently infected animals, but it has been used to study persistently infected permanent cell lines from non-bovine species [181], to biologically characterize an immortalized bovine thyroid cell line [185] and to investigate persistent FMDV infection in an air–liquid interphase (ALI) cell culture model prepared from bovine epithelial tissue from the dorsal soft palate.

### In-vitro models of FMDV pathogenesis

Most primary cells and permanent cell lines used in FMDV research have been selected for maximum susceptibility and permissivity. This makes them very useful for the recovery of infectious FMDV from clinical samples or for the efficient production of FMDV antigen. At the same time, their utility for translatable studies of FMDV pathogenesis is limited because of their dissimilarity with the natural host environment; e.g., their innate immune functions are often significantly impaired and adaptive immune responses are entirely absent. Carefully selected cell culture models, however, can still be useful. Particularly attractive for in-vitro studies is the phenomenon of persistent infection. Its investigation in animals is logistically challenging not least due to the high cost and animal-welfare challenges of keeping ruminants in high containment facilities for the long timeframes involved, and different in vitro models have been proposed to elucidate the molecular mechanisms that support persistent infection [186–188]. A primary cell culture derived from bovine pharynx tissue was persistently infected with FMDV in vitro. Characterization of both the virus recovered from the persistent culture and of the persistently infected cells themselves suggested that the genetic and phenotypic variations arising during persistence reflect a co-adaptation of virus and host cells. Similar to the observations in persistently infected bovine tissues described above, a downregulation of antiviral cytokines was found in the persistently infected bovine pharynx cells [188].

The drawback of cells in culture is that they need to be regularly disrupted and passaged to survive, which is an unnatural process that can induce a variety of artefacts, including substantial alterations of the cell phenotype. To avoid such passages, ALI multilayer cultures of cells from the dorsal soft palate have been developed that remain intact for several months, only requiring a regular exchange of culture media [189]. Using RNA-seq and mass spectrometry to analyze the host response to FMDV infection in this model, Pfaff et al*.* [190] have found stark differences between acute and persistent infection. Acute infection saw the expression of many ISGs leading to strong antiviral activity, whereas persistent infection was characterized by a long-lasting but limited innate antiviral response that was ultimately ineffective at clearing the virus. The small scale of the ALI model allows studies that are very challenging in large animals, e.g. using pharmacological intervention to influence the persistent infection. Similar models using cells of ovine and porcine origin have now been developed. They will be very useful for comparative studies between species that are susceptible to persistent FMDV and those that are not. However, it is essential to recognize that ex vivo systems cannot fully simulate processes in biologically complex organisms. In particular, they omit the host adaptive immune system, which is likely a critical component of the maintenance or clearance of persistent infection [6]. The incorporation of advanced in-vitro models, such as organoids cultured with immune components like T cells or other immune effectors, could provide a more comprehensive understanding of the interplay between FMDV and the host immune response, potentially shedding light on the mechanisms underlying viral persistence.

## Conclusions: persistent knowledge gaps in FMDV pathogenesis

Despite substantial advances in FMDV research through the past decade, there are still many critical knowledge gaps (Table 2) that limit practical improvements in controlling and ultimately eradicating this devastating disease. The gaps span multiple disciplines including epidemiology, virology, immunology, vaccinology and pathogenesis. Although synergistic progress is clearly needed, there is no single discipline that intertwines with all other aspects to the extent of virus-host interactions, i.e. pathogenesis. A comprehensive list of current knowledge gaps pertaining to FMDV pathogenesis is provided in Table 2, and an integrative summary from that list follows below.

**Table 2:** **FMDV pathogenesis gaps.**

Basic pathogenesis
• To what extent does FMDV pathogenesis and clinical presentation differ across diverse breeds of cattle and pigs?
• To what extent is pathogenesis similar across strains of FMDV, particularly SAT serotypes versus others?
• What are the host determinants of immunity, tropism, and virulence; and how are these related to host genomics?
• What are the viral determinants of tropism and virulence?
• What are the sites of primary and persistent infection in goats, Asian buffalo, and susceptible wildlife species?
• How can better understanding of tropism and primary infection be exploited to generate “rationally designed” vaccines?
• How is viraemia established and maintained?
• What unique pathogenesis events occur in gravid females and embryos/fetuses?
• What are the specific virus–host interactions that determine (cardio)myotropism?
• How do pathogenesis events (e.g. shedding and viral evolution) interface with ecological factors to determine geographical range of distinct serotypes and strains of FMDV?
Subclinical neoteric and persistent FMDV infection
• What are the viral and host determinants of establishing and clearing persistent infection at the species and individual animal levels?
• What are the anatomical and cellular sites of persistence in different ruminant species, specifically goats, cervids, and Asian- and African buffalo?
• (How) does vaccine-induced immunity affect persistence?
• Why are ruminants, but not suids susceptible to persistent FMDV infection? And, how might this be exploited to prevent or cure persistence?
• Is non-lytic release of FMDV from infected cells a feature of persistent infection?
• To what extent are persistently infected ruminants a threat to naive animals?
• What are the pathogenesis events and transmission risk associated with neoteric subclinical FMDV infection?
• What is the transmission risk associated with neoterically superinfected carrier animals?
• To what extent are neoterically superinfected carrier animals the source of recombinant FMDV strains and mutational FMDV diversity
Wildlife and transmission
• What wildlife species are capable of becoming FMDV carriers?
• What wildlife carrier species are capable of intra- and/or inter-species transmission?
• How do host species-specific aspects of FMDV pathogenesis relate to transmission competence (as donor or recipient), both within- and between species?
• What is the likelihood that various wildlife species could play a significant role in a natural incursion and/or maintenance of FMDV in previously free regions?

Perhaps the greatest recent advances in pathogenesis work have been the clarification of temporo-anatomic mapping of viral movements through distinct phases of infection in cattle and pigs. However, these breakthroughs are tempered by various practical limitations of these bodies of work. The work in cattle and pigs is complemented by more modest bodies of work in other species, including sheep and African buffalo. However, work on those species has suggested substantial differences and similarities from cattle and pigs that clearly requires more extensive investigation; furthermore, other important domestic (e.g. goats and Asian buffalo) and wild (e.g. wild boar and cervids) species have not been pursued. Furthermore, the vast majority of detailed experimental FMDV studies in cattle and pigs have been based on a very narrow range of host breed- and age groups which, likely, do not accurately reflect the virological, clinical presentation, or transmission dynamics seen in other ages and breeds which are of critical relevance in endemic and outbreak contexts. As examples, indigenous cattle breeds in Asia and Africa are widely reported to be less susceptible to clinical FMD, as are older animals that have received repeated vaccinations; yet, the truth of these suppositions and their purported mechanisms are uninvestigated. Although increasing sources of “big data” describing the host genomic and transcriptomic components of FMDV pathogenesis are becoming available, the analytic tools are still insufficient to enable thorough understanding.

These gaps in characterizations of species-, breed-, and age-specific pathogenesis are important building blocks of basic and translational knowledge, which have their greatest practical relevance when considering how they impact FMDV transmission and epidemiology. Transmission is largely determined by loads of viral shedding by donors and routes of primary infection of recipients, thus the absence of this basic pathogenesis information across species precludes having informed understanding of how the virus could be expected to be maintained within and move between individual animals, groups, and populations. Such information is also missing for the distinct phases of infection with different transmission capacities including preclinical, neoteric, clinical, and persistent forms of infection.

The relevance of knowledge gaps around FMDV transmission are further heightened for the controversial topic of contagiousness during the FMDV carrier state in ruminants. Although transmission from carriers has never been documented experimentally, numerous phenomena surrounding FMDV evolution and control cannot be fully explained without the contributory effects from persistently infected carriers. Specifically, although still unconfirmed, the existence of naturally occurring recombinant FMDVs has only been mechanistically explained by transmission from superinfected carrier cattle. Thus, this proposed source of FMDV diversity from neoteric and carrier ruminants remains unresolved.

Another important category of knowledge gaps in FMDV pathogenesis research exist in the understanding of detailed mechanisms of pathogenesis, spanning both viral and host factors, ranging from a molecular scale to in vivo. Multiple pathogenesis concepts have been incrementally illuminated, such as differences in tissue tropism, viral shedding patterns, and susceptibility to persistent infection, immune responses, and transmission dynamics between different FMDV susceptible hosts. However, the causality of these phenomena remain poorly elucidated. Novel approaches to immunology and gene expression analyses, particularly through NGS approaches have generated a plethora of data; however, much of the information remains at the level of proposed hypotheses. The gaps to be filled should come from the experimental investigations of those hypotheses. The most obvious example is the persistent lack of understanding of the molecular events that enable FMDV to enter and successfully replicate in some host cells but not others. Although canonical and non-canonical receptors have been known for decades, full understanding of FMDV’s determinants of virulence, tropism, and the host cells continuum of permissiveness and prevention remain elusive.

There are several knowledge gaps in pathogenesis of atypical routes of transmission. Further investigation of biological aspects of infection of FMD-susceptible wild species is important for gaining understanding of wildlife-livestock interfaces [112, 113]. Similarly, although environmental contamination and feed-mediated transmission pathways are known to be biologically relevant, systematic quantification through pathogenesis studies would translate to more precise epidemiological interpretation and extrapolation [102, 106].

Addressing these prioritized gaps will advance our understanding of the fundamental concepts of FMDV pathogenesis; however, the real benefit will be derived from the resultant translations to other disciplines of research. For instance, improved quantitation of shedding in multiple species through different stages of clinical and subclinical infection could enhance parameterization of models that could more accurately predict the spread of outbreaks. Similarly, precise understanding of the molecular mechanisms of FMDV persistence could drive creation of novel vaccines that induce sterile immunity, thereby facilitating the viability of vaccinate-to-live policies in FMD free regions. Thus, pathogenesis sits at a crossroads of FMDV research and, similarly, filling the gaps of knowledge in pathogenesis may lead to unanticipated improvements to global FMD control.

## Data Availability

All data generated or analysed during this study are included in this published article and cited publications.
